# P01-009 – 2 years of colchicine IV in intractable FMF

**DOI:** 10.1186/1546-0096-11-S1-A13

**Published:** 2013-11-08

**Authors:** L Kaly, N Boulman, D Rimar, G Slobodin, I Rosner, M Rozenbaum

**Affiliations:** 1 Rheumatology, Bnai Zion medical Center-Haifa Israel, Haifa, Israel

## Introduction

Oral colchicine therapy has been shown effective both in controlling the symptoms and preventing the development of amyloidosis in FMF. However, 5 to 10% of FMF patients are resistant to this conventional therapy.

## Objectives

The objective of this study is to evaluate the efficacy and safety of weekly intravenous colchicine (IVC) adjunct therapy for more than one year.

## Methods

We retrospectively reviewed records of our day-hospitalization from 2007 to 2012 for patients with FMF unresponsive to oral treatment who were treated with supplemental 1mg IVC (120 min infusion) once a week. We tabulated records of clinical events: fever, number of attacks per month and CRP levels before and after at least one year of this treatment. Adverse events were also recorded, and compared with a matched control population of FMF patients only treated with oral colchicine.

## Results

Twelve patients were identified. Two with poor compliance were excluded. Mean age was 39; 70% male. Mean daily dose of oral colchicine was 2.1mg. Mean duration of treatment with IVC was 2.1 years. The number of attacks per month decreased by 68% (4 versus 1.3, before and after respectively, p< 0.008). One patient exhibited an adverse event: deep vein thrombosis of lower extremity.

## Conclusion

Although IVC is a controversial option as treatment for oral-colchicine- non-responsive FMF patients, our study provides evidence of efficacy and relative safety for this modality as long term treatment ,in young FMF patients without comorbidities treated in an academic center, experimented with the use of IVC

## Disclosure of interest

None declared

